# Interstitial Ectopic Pregnancy: The Role of Mifepristone in the Medical Treatment

**DOI:** 10.3390/ijerph18189781

**Published:** 2021-09-17

**Authors:** Guglielmo Stabile, Federico Romano, Giulia Zinicola, Ghergana Alexandrova Topouzova, Giovanni Di Lorenzo, Francesco Paolo Mangino, Giuseppe Ricci

**Affiliations:** 1 Institute for Maternal and Child Health IRCCS “Burlo Garofolo”, 34137 Trieste, Italy; federico.romano@burlo.trieste.it (F.R.); giovanni.dilorenzo@burlo.trieste.it (G.D.L.); francesco.mangino@burlo.trieste.it (F.P.M.); giuseppe.ricci@burlo.trieste.it (G.R.); 2 Department of Medicine, Surgery and Health Sciences, University of Trieste, 34127 Trieste, Italy; giulia.zinicola@burlo.trieste.it (G.Z.); gheri6@gmail.com (G.A.T.)

**Keywords:** ectopic pregnancy, interstitial pregnancy, mifepristone, methotrexate, conservative treatment, medical therapy, fertility sparing

## Abstract

Interstitial pregnancy is defined as the presence of a gestational sac in the most proximal section of the fallopian tube. Management of interstitial pregnancy remains a debated topic. Depending on hemodynamic stability, size of pregnancy, depth of surrounding myometrium, and desires for future fertility, interstitial pregnancy can be managed medically or surgically. We reviewed the literature in December 2020 using keywords “interstitial pregnancy”, “medical treatment”, “methotrexate”, and “mifepristone”. Articles published from January 1991 until 2020 were obtained from databases EMBASE, SCOPUS, and PUBMED. We describe the case of a patient with an interstitial pregnancy that was managed with a total medical approach in August 2020 at Burlo Garofolo Hospital. The patient was asymptomatic and hemodynamically stable, with a high level of serum *β*-hCG (22,272 mUi/mL). We used the combination of methotrexate (MTX) and mifepristone. Medical therapy was effective leading to interstitial pregnancy resolution in 51 days without collateral effects for the patient. We found seven previous cases reported in the literature. Our purpose is to underline the efficacy of medical therapy with systemic multidose MTX associated with a single oral dose of mifepristone and also folinic acid when is present a viable fetus and a high serum *β*-hCG level.

## 1. Introduction

Interstitial pregnancy is defined as a gestational sac implanted in the most proximal section of the fallopian tube (called the interstitial portion) within the myometrium [1]. The term interstitial pregnancy is often used incorrectly as a synonym for “cornual” or “angular” pregnancy. It is important to clarify the difference between these types of ectopic pregnancies, because they are characterized by extremely different rates or morbidity and mortality. The definition of “angular pregnancy” involves the presence of an embryo implanted medial to the utero-tubal junction, in the lateral angle of the uterine cavity. It is not classified as an ectopic pregnancy and generally it does not cause life threatening obstetric complications. Cornual pregnancy denotes a situation in which embryo implants in the cavity of a rudimentary horn of the uterus, which may or may not be communicating with the uterine cavity. It is not an ectopic pregnancy. The term “interstitial pregnancy” refers to an ectopic pregnancy located in the portion of the fallopian tube that penetrates the uterine muscular layer. Management of interstitial pregnancy remains a debated topic. This rare condition is increasing in incidence and accounts for around 2–6.8% of all ectopic pregnancies [2] and constitutes 2–4% of all tubal ectopic pregnancies [3]. Several factors may be involved as risk factors for ectopic pregnancies in general: a previous ectopic pregnancy, the more prevalent pelvic inflammatory disease, pelvic surgery, and assisted reproductive techniques. The increase in tubal sterilization procedures, not always performed properly, can be considered another risk factor for this condition. Moreover, the higher interstitial pregnancies incidence may be due to better diagnosis and improvements in ultrasound [2]. The mortality rate is around 2–2.5%, which is seven times the average for all ectopic pregnancies. The gestational sac implants itself in a thick section of the fallopian tube (averaging 0.7 mm in diameter and 1–2 cm in length) and the layer of overlying myometrium is able to expand much more than the distal portion before rupture [2,3]. Timely diagnosis is the key for successful management of interstitial pregnancies. The most common symptoms of interstitial pregnancy are abdominal pain and vaginal bleeding. A percentage of patients do not have any clinical manifestation [4]. The great spectrum of presentation leads to a difficult diagnosis: the challenge is distinguishing an interstitial pregnancy from an intra-uterine or isthmic pregnancy. Ultrasound represents the most effective diagnostic tool. Sonographically, the demonstration of a rim of myometrial tissue surrounding the eccentrically located gestational sac with an echogenic line extending into the corneal region is highly specific for an interstitial pregnancy [1]. In some cases, the ultrasound localization of blastocyst’s implantation cannot be defined. Magnetic resonance imaging may be used if ultrasound is inconclusive in rolling out the diagnosis of interstitial pregnancy. It is particularly useful in differentiating it from an angular or an intrauterine pregnancy. When the mentioned radiologic tools are not enough in establishing a correct diagnosis, laparoscopic diagnosis is needed [5]. Depending on the hemodynamic stability of the patient, size of pregnancy, depth of surrounding myometrium, and desires for future fertility, interstitial pregnancy can be managed medically or surgically with laparoscopy or laparotomy. Traditional treatment is represented by hysterectomy or cornual resection requiring at the minimum laparoscopic surgery and occasionally laparotomy. Excisional techniques have the potential risk to be complicated by bleeding and additionally leave a scar in an already weakened uterine wall. Other techniques reported in literature include hysteroscopic removal of the interstitial pregnancy under laparoscopic visualization and ultrasound guidance, and uterine artery embolization with systemic treatment [2]. In some cases, an expectant management can be adopted, especially when the patient is asymptomatic and there is a spontaneously declining serum Beta Human Chorionic Gonadotropin (*β*-hCG) level. This gestational strategy allows to avoid chemotherapy and surgical risks. Furthermore, fertility is preserved; however, limited data are available regarding tubal patency and subsequent pregnancy outcomes [5]. Despite the advent of conservative strategies, the most appropriate technique for treatment of interstitial pregnancy and treatment of these patients during subsequent pregnancies remains controversial. In this manuscript we present a case of interstitial pregnancy treated totally medically with the use of methotrexate (MTX) and mifepristone and folinic acid. This medical approach was reported two more times in literature by Gomez Garcia et al. in 2012 and by our research group in 2020 when we have presented two precedent cases successfully treated with this medical protocol at our department [4]. Reviewing literature this is the case of interstitial pregnancy with the highest Beta Human Chorionic Gonadotropin (*β*-hCG) treated with mifepristone, MTX, and folinic acid. We considered all treatment options, both conservative treatments (expectant management, systemic methotrexate, local injection of methotrexate or potassium chloride, and selective uterine embolization) and surgical treatments [6]. We chose this type of therapy considering our previous experience, the gestational age at diagnosis, the patient’s stable hemodynamic state, the size of the mass, the absence of abdominal free fluid, the absence of hepatic, renal and hematological impairment, and the patient’s desire for future fertility.

## 2. Materials and Methods

**Figure 1:**
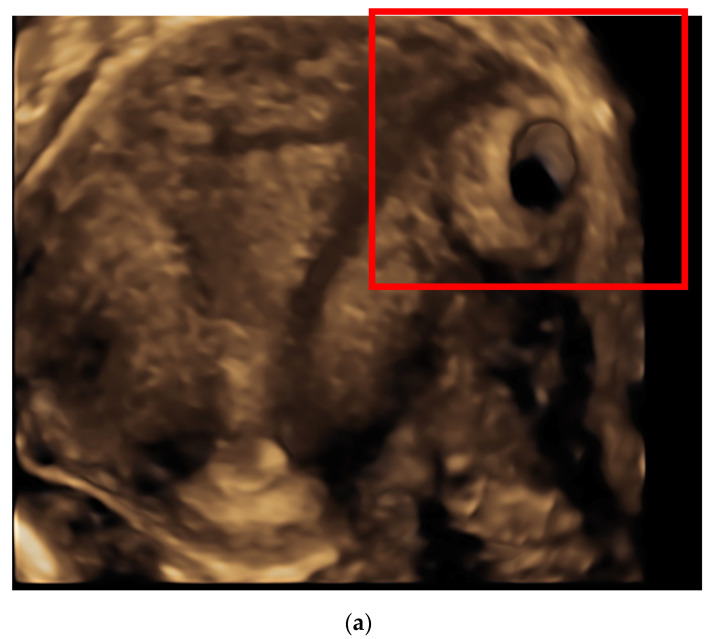

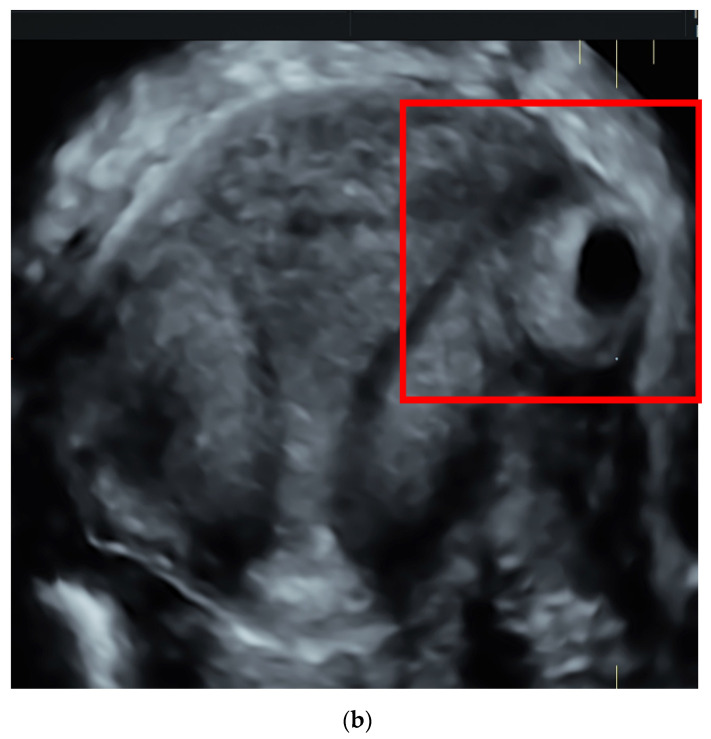
Ultrasound findings of interstitial pregnancy (**a**) A 3-D ultrasonography: an empty uterine cavity with a gestational sac located > 1 cm from the sideward portion of the uterine cavity; the continuation of myometrial mantle around the ectopic sac is clearly viable. (**b**) A 2D-view of the interstitial pregnancy surrounded by a myometrial layer of less than 5 mm.

We present one case of interstitial pregnancy diagnosed and managed with a total medical approach in August 2020 at Burlo Garofolo Hospital. The patient was totally asymptomatic and hemodynamically stable, with high level of serum *β*-hCG (22,272 mUI/mL). The gestational sac with evidence of embryo echoes corresponding to 7 weeks of gestational age and heart beating was detached on a routine transvaginal ultrasound (TVUS) in the interstitial portion of the right tuba. The woman had a previous ectopic pregnancy of the right tuba 3 months before, treated laparoscopically with a mono-lateral salpingectomy. An experienced operator performed a TVUS, which showed an empty uterine cavity, a gestational sac (diameter 40 mm) located > 1 cm from the sideward portion of the uterine cavity, surrounded by a myometrial layer of less than 5 mm. According to Timor-Tritsch’s criteria (1992) [7] the interstitial pregnancy diagnosis was confirmed (Figure 1). It was also revealed the interstitial line between the pregnancy and the lateral edge of the endometrial cavity [8]. Considering clinical presentation and desire for future pregnancy, it was chosen a fertility sparing treatment based on the combination of a single dose of mifepristone 600 mg orally, the multiple dose protocol consisting of the intramuscular injection of 1 mg/kg of MTX and 0.1 mg/kg of folinic acid. Alternate daily doses of each were administrated four times and *β*-hCG declined > 15% from previous measurement (Table 1). Serum *β*-hCG levels dropped down to 6100 mUi/mL in 12 days. The patient was discharged and underwent serial checks weakly, until *β*-hCG became undetectable. Medical therapy was effective leading to interstitial pregnancy resolution in 51 days without collateral effects for the patient. A residual interstitial mass, as a heterogeneous area with persistent vascularity has been reported at TVUS, despite complete *β*-hCG resolution. A literature search was carried out in December 2020 using the keywords “interstitial pregnancy”, “medical treatment”, “methotrexate”, and “mifepristone”. Articles that were published from 1991 until 2020 were selected from databases Embase, Scopus, and PUBMED. One article was excluded from our analysis: the medical treatment using local MTX and oral mifepristone was combined with the Laparoscopic salpingocentesis [9]. The institutional review board (RC 08/2020) approved this descriptive study in April 2020. The patient signed an informed consent before treatment. Permission for the publication was taken in accordance with the 1964 Helsinki Declaration and its later amendments or comparable ethical standards.

## 3. Results

In the present literature we found four studies reporting seven previous cases of interstitial pregnancies successfully treated using the combination of MTX with a single oral dose of mifepristone 600 mg, and one with a dose of mifepristone 200 mg (Table 1) [4,11,12]. Patients’ clinical characteristics, ultrasound- and laboratory findings, and type of treatment adopted are presented in Table 1. The mean gestational age at diagnosis of cervical pregnancy was 6 weeks + 4 days and the mean value of beta-HCG at diagnosis was 7683.85 mUI/mL, ranging from a minimum value of 594.8 mUI/mL to a maximum value of 22,272 mUI/mL. Our case of interstitial pregnancy is characterized by the higher *β*-hCG level currently presented in the literature. The earliest diagnosis regarded a patient at 5 weeks + 1 day of gestation, the latest diagnosis was performed at 8 weeks + 3 days. In three cases (*n* = 3; 37.5%) an embryo was present inside the gestational sac; embryo heartbeat was present in two of the three cases. Two patients (*n* = 2; 25%) needed more than one dose of MTX. Two women had documented risk factors for ectopic pregnancies: the first one had undergone a monolateral salpingectomy for a previous tubal pregnancy and the second one had three past interruptions of pregnancies with hysterosuction and curettage.

All the seven cases reported had no side effects and treatment with the combination of MTX and Mifepristone lead to the resolution of pregnancy.

## 4. Discussion

Interstitial pregnancies are a rare type of ectopic pregnancy associated with an increased risk of severe hemorrhage and maternal morbidity. Management of interstitial pregnancies remains a debated topic, with no clear guidelines on the best approach. Advances in TVUS and availability of quantitative *β*-hCG allow diagnosis of interstitial pregnancy at an earlier gestational age [4]. TVUS has been reported to have a high sensitivity at 6.9 weeks’ gestation. On the contrary to what one might believe, 3D-US is not more precise for diagnosis of interstitial pregnancies compared to 2D-US. The diagnosis of interstitial pregnancy by ultrasound is determined by three criteria: the gestational sac is implanted outside the uterine cavity; the interstitial part of Fallopian tube is seen adjoining the lateral aspect of the uterine cavity and the gestational sac is surrounded by myometrium [2]. There are no data in literature of particular serum *β*-hCG trends that can guide diagnosis and allow to differentiate intrauterine pregnancies from ectopic embryo implantations [2]. The treatment should be personalized considering the obstetric history of the patients, the gestational age at the diagnosis, and their desire for future pregnancies [3]. Expectant management could be a first-line approach in selected asymptomatic patients at an early presentation but this therapeutic choice is burdened by increased morbidity and mortality. In hemodynamically unstable patients or in those where medical therapy has failed, surgery is the treatment of choice. Laparoscopic treatment may be considered the gold standard currently for a surgical approach [12,13]. Laparoscopic cornual resection is well described and requires advanced laparoscopic skills. Laparoscopic resection is associated with risk of bleeding and has the disadvantage of causing a permanent uterine scar. It represents a potential risk for uterine rupture in future pregnancies. This is the reason why possible future pregnancies should be considered at risk and monitored by serial ultrasound scans. Another strategy of treatment is represented by uterine artery embolization under fluoroscopic and 3D-ultrasound guidance. It should be considered that, in women treated with this technique, several cases of endometrial atrophy and adverse effect on fertility are described [14]. To date it remains experimental until more information can be obtained regarding safety and efficacy especially in childbearing age [5]. Medical treatment for interstitial pregnancy appears to be a safe and effective option in carefully selected cases. The decision to proceed with medical management depends on the clinician’s discussion with the patient. Medical treatment is based especially on the use of methotrexate. However, other chemotherapeutic agents (actinomycin D and etoposide) have been experimentally injected into the gestational sac with success. More data regarding their safety and efficacy are needed. According to the literature, one of the most effective local agents in the case of a heterotopic pregnancy, not only of the interstitial type, with a viable embryo, is represented by potassium chloride 20% [5]. MTX is the more widely used chemotherapeutic agent well established as a reasonable non-surgical treatment for selected ectopic pregnancies and it was first cited in 1982 [15,16]. MTX is a folinic acid antagonist that interferes with DNA synthesis in rapidly dividing cells at the implantation site. There is not a specific cut-off value below which the drug should not be used, but with high basal serum *β*-hCG levels the success rate is extremely variable. A recent review reports no statistically significant difference between the two routes of administration: it could be injected systemically by the intramuscular or intravenous route [2], or locally, close or into the gestational sac, using laparoscopic, ultrasonographic, or hysteroscopic guidance. Local injection of MTX reported a success rate between 91–100%, but technical expertise and additional costs are required. We support the hypothesis that TVUS evaluation of perilesional vascularization could influence the gynecologist’s choice about mode of administration (local or systemic). It can be supposed that a higher vascularization of the peripheral portion of interstitial pregnancy could indicate a better outcome with the use of systemic therapy due to two aspects. Firstly, it reduces the risk of bleeding during an eventual invasive procedure. Secondly, the large vascularization increases drug capacity to reach the trophoblast [4]. Systemic MTX can be administered following single-dose, double-dose, or multiple-dose protocols. In the single-dose protocol only one dose of intramuscular MTX (50 mg/m^2^ body surface area) is administered. The serum *β*-hCG levels are observed on day 4 and on day 7. If the serum HCG level reduction is less than 15%, a second dose of MTX is required [17]. The double-dose regimen provides for the administration of two methotrexate doses on day 0 and on day 4 [18]. The multiple dose protocol consists of an intramuscular injection of 1 mg/kg of MTX with 0.1 mg/kg of folinic acid 24 h later to reduce side effects. This regimen is continued on alternating days until the *β*-hCG level drops by 15% over two days. Up to four doses may be given to one patient, but not all four doses have to be given [19]. With multiple doses of MTX for interstitial pregnancy, success rates of 66 to 100 percent have been reported [10,20]. In 2017, Yang et al. [21] published a systematic review and meta-analysis study for comparison of multiple-dose and double-dose versus single-dose administration of MTX for the treatment of EP. They identified six randomized controlled trials through database searching, and after the analysis, they observed that the overall success rate of multiple-dose protocol was similar to the single-dose protocol. A previous meta-analysis by Barnhart et al., which included 26 case series, found that the multiple-dose regimen was slightly more effective than single-dose regimen [20]. No studies, however, directly compared the two medical regimens. Considering the high serum BHCG level (the highest value presented in literature), the presence of a viable embryo and the evidences in literature slightly in favor for multiple dose regimen we opted in this case for using it.

MTX can be used also in addition to a surgical procedure, in order to minimize bleeding [22]. For the first time in the literature, Perdu et al. in 1998 reported the combination of mifepristone and MTX assuming that the synergic action of the two drugs could induce the trophoblast lysis more rapidly than MTX alone [23]. To understand the activity of mifepristone on pregnancy, one must first have an understanding of the role of progesterone in pregnancy development. Progesterone is required for both pregnancy development and maintenance. After ovulation, the corpus luteum secretes progesterone to create a secretory endometrium appropriate for embryo implantation. Once implantation has occurred, progesterone suppresses uterine contraction. Continuation of pregnancy is dependent on luteal progesterone until placental secretion of progesterone will be sufficient [24]. Mifepristone is a steroidal antiprogestogen drug that can competitively binds the progesterone receptor. This combination leads to a degeneration of the cell of decidua. It also blocks the corpora lutea [25]. Mifepristone relative affinity for the human uterine progesterone receptor is two to 10 times that of progesterone. In the literature there is a case of interstitial pregnancy with *β*-hCG levels less than 5000 mIU/mL successfully treated with a single dose of mifepristone 200 mg (the only case with this type of dosage) and with an IM injection of MTX 50 mg without surgical intervention [26]. We chose to use mifepristone 600 mg on the basis of our previous experience and the presence in the literature of more previous cases of interstitial pregnancy successfully treated with this dosage. Mifepristone has not been shown to be effective in tubal pregnancies. We hypothesize that its efficacy in the interstitial pregnancies is due to the presence of the endometrium, and, therefore, of progestin receptors, in the initial portion of the tube. The use of an antiprogestogen agent such as mifepristone can interfere with the pregnancy development in this particular site thus enhancing the effect of MTX. Garbin et al. demonstrated that the adjunct of mifepristone does not increase the efficacy of MTX when progesterone level was >10 ng/L [27]. This observation reinforces the concept that an evaluation of pregnancy vascularization, even if subjective, could be the leading point to choose a combined therapy. A higher vascularization could suggest the presence of a wider syncytiotrophoblast and a consequent higher progesterone secretion. This aspect could be the cause of a minor efficacy of the therapy with mifepristone. Syncytiotrophoblast plays the most important role in maintaining pregnancy by directly contacting the endometrium for gas exchange. It also secretes human placental lactogen to absorb the nutrients needed by the fetus from the mother, secretes *β*-hCG to maintain the corpus luteum of the ovary, and regulates the secretion of estrogen and progesterone [28]. The combination of MTX and mifepristone appears to be a good option for interstitial pregnancy treatment, although more data are required. MTX is not free from side effects, including myelosuppression and digestive symptoms, such as mouth sores. The worst complication is failure to induce pregnancy shutdown, with appearance of bleeding, which could be life threatening. Close follow-up in patients medically treated is advised. It is important to remember that an adequate medical counseling is necessary before treatment and before a subsequent conception, also if there is an unknown risk of uterine rupture in a future pregnancy [20]. Clinical management of interstitial pregnancy remains a debated topic and a challenge for gynecologist who have to face this pathology in absence of guidelines.

## 5. Conclusions

Interstitial pregnancy is a rare disease, but we can consider it as an emerging problem in light of the growing increase in assisted reproductive techniques, which represents an important risk factor. In this paper, we present a case of interstitial pregnancy with a viable embryo and the higher *β*-hCG level currently present in the literature. We support the hypothesis of the safety and feasibility of the combination of MTX and mifepristone for this rare condition, although in case of high serum *β*-hCG considering clinical presentation, obstetric history, gestational age at the diagnosis, and desire for future pregnancies, but treatment should be always personalized. Further studies as clinical trials or prospective studies from multiple centers are required to establish which is the best approach for interstitial pregnancy management. Women need to be counseled that after having an interstitial pregnancy and corresponding treatment, their future risk of ectopic pregnancy is increased, and an early ultrasound scan is paramount in future pregnancies.

## Figures and Tables

**Table 1:** Management of interstitial pregnancy using systemic MTX combined with Oral mifepristone.

Reference	Pregnancy Hystory	GA	Embryo (CRL mm)	FHB	Basal *β*-hCG mUi/mL	Management
Current Case	G2P0 (1 Previous EP treated by LPS: monolateral salpingectomy)	7 weeks	9	+	22,272	Mifepristone 600 mg + MTX 1 mg/kg + 0. 1 mg folinic acid (four doses)
Case 1 Stabile G. et al. 2020 [4]	G1P0	7 weeks	3.6	+	19,397	Mifepristone 600 mg + MTX 1 mg/kg + 0.1 mg folinic acid (single dose)
Case 2 Stabile G. et al. 2020 [4]	G2P1	6 weeks + 6 days	no embryo	−	2664	Mifepristone 600 mg + MTX 50 mg/m^2^ of body surface (double dose)
Case 3 Gomez Garcia et al. 2012 [8]	G2P1	8 weeks +3 days	no embryo	−	3724	Mifepristone 600 mg + IM MTX 50 mg/m^2^ of body surface (single dose)
Case 4 Gomez Garcia et al. 2012 [8]	G4P0 (3 VIPs)	6 weeks + 3 days	6	−	4116	Oral mifepristone 600 mg + IM MTX 50 mg/m^2^ of body surface (single dose)
Case 5 Stabile G. et al. 2020 [9]	G3P1	6 weeks	no embryo	−	6579	Mifepristone 600 mg + MTX 50 mg/m^2^ of body surface (single dose)
Case 6 Stabile G. et al. 2020 [9]	G3P1	5 weeks + 3 days	no embryo	−	2124	Mifepristone 600 mg + MTX 50 mg/m^2^ of body surface (single dose)
Case 7 Karki et al. 2015 [10]	G1P0	5 weeks + 1 day	no embryo	−	594.8	Oral mifepristone 200 mg + IM MTX 50 mg/m^2^ of body surface (single dose)

*β*-hCG = Beta Human Chorionic Gonadotropin; MTX = methotrexate; CRL = crown rump length; IM = intramuscular; FHB = fetal heartbeat; G = gravidity; P = parity; EP = ectopic pregnancy; LPS = laparoscopy; VIP = voluntary interruption of pregnancy. No other therapeutics strategies needed in any cases.

## Data Availability

The original contributions presented in the study are included in the article, further inquiries can be directed to the corresponding author.
